# Identifying facilitators and barriers to integrated and equitable care for community-dwelling older adults with high emergency department use from historically marginalized groups

**DOI:** 10.1186/s12939-023-01900-y

**Published:** 2023-05-19

**Authors:** Krystal Kehoe MacLeod, Karyle Nama Flores, Kavish Chandra

**Affiliations:** 1 Bruyère Research Institute, Ottawa, Ontario Canada; 2 Faculty of Medicine, University of Ottawa, Ottawa, Ontario Canada; 3 Horizon Health Network, Saint John, New Brunswick Canada; 4 Faculty of Health Sciences and Nursing, University of New Brunswick, Saint John, New Brunswick Canada; 5 Faculty of Medicine, Dalhousie University, Saint John, New Brunswick Canada

**Keywords:** Emergency department avoidance, Frequent users, Health services accessibility, Mixed methods, Older adults, Unmet care needs

## Abstract

**Background:**

High rates of emergency department (ED) use by older adults persist despite attempts to improve accessibility of appropriate and comprehensive care. Understanding the drivers of ED visits from the perspective of older adults from historically marginalized groups could help reduce ED use by patients with needs that are preventable or could have been treated in a more appropriate setting. This interpretivist, feminist study aims to explore the unmet care needs of older adults (age 65 +) with high ED use and belonging to historically marginalized groups to better understand how social and structural inequities reinforced by neoliberalism; federal and provincial governance structures and policy frameworks; and regional processes and local institutional practices, shape the experiences of these older adults, particularly those at risk of poor health outcomes based on the social determinants of health (SDH).

**Methods/design:**

This mixed methods study will employ an integrated knowledge translation (iKT) approach, starting with a quantitative phase followed by a qualitative phase. Older adults self-identifying as belonging to a historically marginalized group, having visited an ED three or more times in the past 12 months, and living in a private dwelling, will be recruited using flyers posted at two emergency care sites and by an on-site research assistant. Data obtained through surveys, short answer questions, and chart review will be used to compile case profiles of patients from historically marginalized groups with potentially avoidable ED visits. Descriptive and inferential statistical analyses and inductive thematic analysis will be conducted. Findings will be interpreted using the Intersectionality-Based Policy Analysis Framework to identify the interconnections between unmet care needs, potentially avoidable ED admissions, structural inequalities, and the SDH. Semi-structured interviews will be conducted with a subset of older adults at risk of poor health outcomes based on SDH, family care partners, and health care professionals to validate preliminary findings and collect additional data on perceived facilitators and barriers to integrated and accessible care.

**Discussion:**

Exploring the linkages between potentially avoidable ED visits by older adults from marginalized groups and how their care experiences have been shaped by inequities in the systems, policies, and institutions that structure health and social care provision will enable researchers to offer recommendations for equity-focused policy and clinical practice reforms to improve patient outcomes and system integration.

## Background

Older adults are clear in their preference to age in their homes and communities for as long as they wish and are able [1]. They have identified timely access to appropriate programs and supports for health care and daily living as fundamental to successful aging in place [2]. ‘Aging in place’ refers to policies that aim to keep older adults out of facility-based care, such as alternate level of care beds in hospitals or nursing homes, by providing them with the health and social supports and services they need to live safely and independently in their home or community [3]. The prioritization of client choice is a major factor influencing the push for aging in place [4]. Additionally, relocating care for older adults from publicly funded institutions into private homes or community settings is seen as an opportunity for cost savings by governments [1, 5].

It is common for older adults aging in their homes or communities to suffer from combinations of multiple chronic conditions; cognitive, functional and mental health impairments; drug interactions; social vulnerabilities and/or frailty [6–8] making them frequent users of many different health and social services [6], including emergency departments [9]. These patients often have substantial experience transitioning through multiple points of care involving many patient-provider interactions [10] at the interfaces of home, community, primary care, and/or hospital [11]. Care transitions occur when the responsibility for a patient’s care is transferred between health and social care providers working across different settings and sectors [12]. Providing good quality, continuous, accessible, and appropriate care to older adults with complex care needs who are aging in place is a complex undertaking for many reasons, including: care transitions in this population are a high-risk scenario for patient safety [13]; community care is chronically underfunded [14]; care work in private dwellings is particularly precarious employment [15]; care provision for this population relies heavily on the unpaid labour of spousal, family, and friend care partners [16, 17]; and issues of fragmentation disrupt and complicate care delivery [18]. Fragmentation refers to components of care systems that “function in silos” [19], such as the separation of health care from social care in organizations, service delivery, and funding mechanisms. Fragmentation can also mean a lack of coordination between those responsible for care; gaps in or the duplication of services and infrastructure across levels or settings; or care that is provided in an inappropriate location [20]. Older adults experience fragmentation in the form of barriers to care; interactional issues among providers and across settings; and unmet decisional needs regarding possible care options [6], all of which can negatively impact their care experiences and impede their ability to successfully age in place. Although the increasing importance of integration to health and social care delivery systems for older adults has not escaped scholarly and policy attention, there remains a gap in equity-focused research exploring the lived experiences of older adults from historically marginalized groups (e.g. sex/gender, LBGTQ2S + , indigeneity (First Nation, Metis and Inuit), race/ethnicity, (im)migration status, and income-level) as they navigate the complexities of the health and social care systems [21] particularly within the current neoliberal context. Neoliberalism is the dominant political and economic ideology, governance structure, and policy toolkit that promotes the use of business solutions to health management and public policy problems [22].

Emergency department (ED) visits have been shown to be a good proxy of health and social services usage [23]. Canadians, in general, appear to use EDs more frequently than people in other Commonwealth countries [24] and, in many jurisdictions, the number of emergency department visits attributable to frequent users is increasing [25]. Patients with complex care needs have particularly high utilization rates of episodic care [26]. It has been determined that as many at 20% of emergency visits could be dealt with more efficiently in settings other than EDs [24], yet high rates of ED visits in Canada persist despite attempts to improve accessibility of appropriate and comprehensive care [7]. Avoidable ED visits occur when patients present at the ED with needs that are preventable or could have been treated in a more appropriate setting [24]. Compared with respondents from other countries, Canadians are more likely to report visiting the ED for a condition that might have been treated in a different care setting: 47% chose to seek care in the ED because they could not get an appointment with a primary health care provider; 38% felt the ED would give them the best care for their condition; and 7% said they were not aware of other settings they could use [24]. This raises concerns about poor care management [27], increased costs to the health care system [24, 28], and possible reductions in continuity of care [29].

Understanding the drivers of potentially avoidable ED use is an essential component in the effort to address the unmet care needs of older adults with complex care needs as they age in place in the Canadian province of New Brunswick. New Brunswick is Canada’s only officially bilingual province with a population of 800,000 in 2022 [30]. Like the rest of Canada, New Brunswick has a publicly funded health care system governed by national legislation but delivered by the provincial government. Deinstitutionalization, operationalized as “aging in place” or “aging at home”, is a primary policy objective of health care restructuring initiatives in New Brunswick. In line with the province’s Aging Strategy (2017), New Brunswick’s Extra-Mural Program and Long-Term Care Services offer older adults in the province a variety of health and social care services, respectively, aimed at assisting them to age in place. While acute and primary care in New Brunswick are publicly funded and delivered, home care in the province is delivered through complicated networks of programs, organizations, and care providers in governments, not-for-profits, for-profits, charitable agencies, communities, and households. Certain types of home care are publicly funded, while others are means-tested.

According to the 2017 New Brunswick Health Council’s Primary Health Survey (PHS), 42% of New Brunswickers reported needing home care services to help them remain at home but not receiving them [31]. The most common types of unmet home care needs were home support services, such as housekeeping, meal preparation, bathing, and shopping [31]. When older adults with complex care needs encounter barriers to care, it can contribute to poorer health outcomes and higher mortality rates while generating considerable costs to the health and social services system [32, 33] through health care services overuse, underuse, or misuse [6]. Moreover, members of historically excluded groups are known to be at greater risk of poor health outcomes based on the social determinants of health such as gender, race/ethnicity, and income level [34]. For example, women experience disproportionately more unmet care needs than men [35, 36] and patients from low-income neighbourhoods and rural communities are more likely to experience challenges accessing appropriate and continuous health care [37, 38]. Health inequalities can contribute to both differences in unmet care needs [39, 40] and higher emergency department use by marginalized groups [41, 42], defined in this study in relation to equity considerations of sex/gender, LBGTQ2S + , indigeneity (First Nation, Metis and Inuit), race/ethnicity, (im)migration status, and income-level.

When compared to other high-income countries with similar per capita spending levels, Canada’s health system underperforms in domains such as access, equity, and health outcomes [43]. A nuanced understanding of the contextual factors driving the correlation between unmet care needs and high ED utilization rates, rooted in the lived experiences of older adults with complex care needs, is key to building stronger and more equitable health and social care delivery systems that are better equipped to address inequalities in access to prevention and care; socioeconomic and ethno-racial inequities; and the lack of coordination across federal, provincial, and local health care and social care organizations. With patients in the top 3% of emergency department utilization accounting for 30% of health care costs [23], and costs increasing with persistent frequent use [44], better understanding these connections offers benefits in both improved equity and efficiency.

With important research emerging in ED-to-community transitions for older adults in North America [45, 46] and global recognition that efforts to integrate care are interconnected to ED avoidance and can lead to reduced ED admissions in the longer-term [47], there is a widespread consensus that improving care for older adults requires better integration of health and social care services [48–50]. Research using an intersectionality lens can help address the existing gap in equity-focused research by seeking to better understand the experiences of older adults with complex care needs and high ED use within the broader social, political, and economic contexts where inequitable structures of power and privilege continue to shape access to care for marginalized groups [51–53]. Intersectionality is a way of understanding and analyzing complexity in the world, in people, and in human experiences. This theoretical lens allows people’s lives and the organization of power in society to be understood as being shaped by many axes of social division that work together and influence each other [54]. In particular, the Intersectionality-Based Policy Analysis (IBPA) Framework will be used in this study to capture and respond to the multi-level interacting social locations, forces, factors, and power structures that shape and influence health and care experiences [55]. Building on other Integrated Knowledge Translation (iKT) work [56], the IBPA framework will help the research team keep considerations of health equity at the forefront of the analysis by focusing on how inequalities in power and privilege of certain individuals and groups in relation to each other impact the lived experiences of older adults from marginalized groups as they navigate care systems. The use of this intersectional lens will complement the iKT approach [57, 58] used in this project by prompting the research team to engage with the lived experiences of knowledge users in ways that permit the synthesis of an evidence base that can be used to affect health care practice and guide policy and process reform at both government and institutional levels [59]. In line with this project’s design, the IBPA framework sees the involvement of knowledge-users as integral in the project team, and patient partners will be meaningfully engaged in all stages of research from project design through to dissemination.

This project will begin at the macro-level by considering the continuing impacts of neoliberalism on shaping care systems in ways that impact the lived experiences of older adults. Next, the impacts of governance structures such as national and provincial level legislation and regulations, funding and resourcing structures, and the division of powers regarding service delivery will be explored. Third, it will look at how meso-level factors such as specific institutional processes and practices shape the delivery of health and social care services to older adults aging in place. Research that places the patient experience at the forefront of the analysis is needed to develop a better understanding of how macro- and meso-level considerations of governance, funding, organization, and delivery structures can reinforce or challenge structural inequities in ways that shape the micro-level care experiences of older adults with complex care needs from vulnerable groups. Making connections across levels of analysis can inform decision making regarding where care services, institutions, and providers interface well from the perspectives of patients and families and where and why disconnects continue to create barriers to equitable access to care for older adults with complex care needs who are aging in place.

This study aims to address the following research questions:

1. How are the unmet health and social care needs of community dwelling older adults (65 + years) from historically marginalized groups with high ED use connected to potentially avoidable ED visits?
2. How have (1) neoliberalism as a dominant ideology; (2) federal and provincial governance structures and policy frameworks; and (3) regional processes and local institutional practices, shaped the care experiences of older adults with high ED use who self-identify as a member of a historically marginalized group, as part of their journey to age in place?

## Research objectives

Poor health in aging populations, combined with pre-existing health inequalities experienced by members of historically marginalized communities (e.g. sex/gender, LBGTQ2S + , indigeneity (First Nation, Metis and Inuit), race/ethnicity, (im)migration status, and income-level), underscore the need for equity-focused health research to better understand how the unmet care needs of these older adults drive potentially avoidable ED use. To this end, the project has four objectives:

1. To identify the unmet care needs of community dwelling older adults (age 65 +) with high ED use.
2. To better understand how these older adults and their family care partners perceive these unmet care needs as connected to their current ED visit.
3. To learn more about how intersecting social relations of inequality (e.g. sex/gender, LBGTQ2S + , indigeneity (First Nation, Metis and Inuit), race/ethnicity, (im)migration status, and income-level) are associated with older adults’ unmet care needs and potentially avoidable ED visits.
4. To provide insight into how the neoliberal paradigm; federal and provincial governance structures and policy frameworks; and regional processes and local institutional practices, shape the lived experiences of older adults from historically marginalized groups leading them to seek out care from EDs as part of their journey of aging in place.

## Methods/design

This interpretivist, feminist, integrated knowledge translation (iKT) study will use a patient-oriented research approach by engaging patients as research partners in mixed-methods research that can be used to improve both patient and system outcomes. The researcher team will conduct a 2-phase mixed methods study [60, 61], starting with a quantitative phase (phase 1) to answer objectives 1 and 2, followed by a qualitative phase (phase 2) for objectives 3 and 4. This design is well adapted to answer research questions addressing complex systems in varied and dynamic contexts, allowing for in-depth analysis of each case, and opportunities for comparison (objective 4). This study will identify the unmet care needs of greatest priority as identified by older adults and their family care partners from diverse and varied backgrounds; be collected through meaningful consultation with patients and family care partners; and be disseminated to clinicians, health administrators, and government decision-makers to inform health system reform for the benefit of older adults with complex care needs and high ED use and their families. A plan for meaningful, safe, and inclusive patient engagement has been developed including the establishment of an Advisory Council comprising researchers, a clinician, a patient, and a family care partner, to oversee project milestones.

### Phase 1

#### Sampling of patients

Following research ethics board approval, research participants will be recruited from the large urban emergency department at Saint John Regional Hospital and a community urgent care clinic in Saint Joseph’s Hospital. These are the main sites where residents of Saint John, New Brunswick, access emergency care. A research assistant will post recruitment flyers in each ED. If patients or family caregivers are interested in learning more about participating in the study, they can remove a contact information tab from the bottom of the flyer or use the QR code to contact the principal investigator (PI). A research assistant will also be present on alternating days at each site to actively recruit participants in the ED waiting room following receipt of consent to be contacted via a member of the care team. Informed by Hudon et al*.*’s study of a similar population in comparable ED settings, including in New Brunswick, [62], we are seeking to recruit 30 patient participants at each ED site (*n* = 60) and 30 family care partner participants (*n* = 60) with a good mix of respondents from diverse groups, including participants representing different sexes/genders, race/ethnicity groups, and income levels, in line with Coleman et al*.*’s recommendation to include more diverse populations in future studies on care transitions and rates of hospitalization [63]. Maximum variation sampling will be used if required to ensure diversity in patient respondents.

#### Research objective 1

To identify the unmet care needs of community dwelling older adults (age 65 +) with high ED use.

The PI will virtually administer an adapted version of the New Brunswick Health Council’s Primary Health Survey (PHS) 2020 as a needs assessment tool to the older adults with complex care needs and high ED use who consent to participate. A health needs assessment is a systematic method for reviewing the health issues facing a population, leading to agreed priorities and resource allocation that will improve health and reduce inequities [64]. The PHS is a self-reported questionnaire that will collect data in key areas including: demographic information, self-reported health; health care model; primary care providers; utilization and care experiences (including ED use); specialist visits; home care; chronic conditions; overall care experiences; wellness/prevention; and difficulties in access. French and English-language versions will be available. The required time to complete the questionnaire will be about 15 min.

#### Research objective 2

To better understand how these older adults and their family care partners perceive unmet care needs as connected to their current ED visit.

Dart and Davies' Most Significant Change (MSC) technique [65] will be adapted for use in this project by adding two open-ended short answer questions to the PHS used in *Objective 1*. MSC is a qualitative, story-based method for gathering data that can be embedded into other research methods, facilitates triangulation and lends itself well to knowledge translation [65]. The crux of MSC is to ask research participants to tell a story of significant change that led to their current situation. For this project, the PI will prompt each patient and the family carer partner who accompanied them to the ED (when appropriate) to respond to two questions. For patients: 1) What about your care needs changed leading you to be in the ED? 2) Why is the change you described important? For family care partners: 1) What about the patient’s care needs changed leading them to be in the ED? 2) Why is the change you described important? These short answer questions will be administered virtually by the PI following the PHS for patients or during a separate call for family care partners and responses will be recorded and transcribed. The required time to respond to the short answer questions is about 5 min.

### Phase 2

#### Research objective 3

To learn more about how intersecting social relations of inequality, specifically sex/gender, LBGTQ2S + , indigeneity (First Nation, Metis and Inuit), race/ethnicity, (im)migration status, and income-level, are associated with older adults’ unmet care needs and potentially avoidable ED visits.

Extending previous work on potentially avoidable visits to NB EDs [66] the research team will categorize de-identified versions of the emergency department charts for each patient participant in Objective 1 as “not avoidable” or “potentially avoidable” using a predetermined set of criteria developed in collaboration with the ED physician on the project’s advisory council. In cases where the chart classification is unclear, the ED physician will review and make a professional judgement.

Next, demographic data from Objective 1 will be used to identify the subset of patients in this study who self-identified as belonging to a historically excluded or marginalized group. These demographic data will be linked to the corresponding responses to the adapted PHS questionnaire, short answer responses to the MSC questions, and emergency department chart data from the ED visit to create a de-identified case file for each patient who qualifies as an older adult with complex care needs with (1) a potentially avoidable ED visit, and (2) at greater risk of poorer health outcomes based on social determinants of health related to equity considerations.

#### Research objective 4

To provide insight into how the neoliberal paradigm; federal and provincial governance structures and policy frameworks; and regional processes and local institutional practices, shape the lived experiences of older adults from historically marginalized groups leading them to seek out care from EDs as part of their journey of aging in place.

Using interpretive description [67], the PI will interview patients (*n* = 10) from the subset of patients identified in Objective 3 and the family care partner who accompanied them to the ED (where appropriate) (*n* = 10) in virtual, telephone, or in-person semi-structured interviews six months after their ED visit. These interviews will be used both as a form of member checking to determine if the study’s preliminary findings from objectives 1–3 are validated by research participants as resonating with their lived experience or contested [68]. The interviews will also be used to discuss the findings in depth and to collect participant stories and examples illustrating if/how they understand social and structural inequities as having shaped their decision to seek care from EDs specifically and their experiences accessing care more broadly during their journey to age in the right place. The researcher will also conduct semi-structured interviews with a selection of health care professionals (*n* = 5) including members of the care team within the EDs and the primary care providers of some of the patients participating in the study, to collect their feedback and ensure that their perspectives are represented.

### Rigour, trustworthiness and data analysis

Statistical analysis of the PHS data in Objective 1 will focus on: perceived health status, health service delivery, and experiences with health services. Overall group data will be analyzed and comparisons will be made between demographic groups within the sample. Data will be entered into SPSS Statistics. Summary statistics will be calculated using descriptive and frequency functions and group comparisons made using independent group t-tests and analysis of variance (ANOVA) models. Group differences in categorical data will be performed using chi-square independence tests. Confidence intervals and effect sizes will be reported. This dataset will also be compared with the province-wide dataset from the PHS 2020 using the same statistical tests.

For qualitative data analysis of the short answer questions, reflexive thematic analysis [69] with inductive coding [70] for understanding influences related to how people respond to events [71] will be used. The analysis will involve generating initial codes and then searching for, reviewing, defining, and naming themes that represent responses within the dataset [69]. The data will be managed using MAXQDA software.

“Mixing” data from Objectives 1 and 2, the PI will compare and contrast case studies developed in Objective 3 [72]. The Intersectionality-Based Policy Analysis Framework will be applied to the data to help researchers identify and interpret intersections between unmet care needs, potentially avoidable ED admissions, structural inequalities, and the social determinants of health. Using an intersectionality lens to interpret the case studies will facilitate an exploration of the complexities of how equity considerations have shaped patients’ experiences of unmet care needs and how structures of power and privilege may have impacted their interactions with the care system and their ability to access integrated care.

Patient partners and the ED physician on the project’s Advisory Council will review the draft interview guides for Objective 4 to ensure their relevance. The interview guide will be pilot tested with a patient partner and refined as required. This type of collaboration will increase the trustworthiness and credibility of qualitative findings [73, 74] and empower patient and provider voices and perspectives in the study’s data collection and analysis. To further support rigour, research assistants and the PI will engage in reflexive journaling and memo-writing during the recruitment and interview phases of the project which will be incorporated in the qualitative analysis. The research assistants involved in the project will meet regularly with the PI and will jointly produce a reflexive paper on their experiences of recruitment and data collection to help the team engage in constant reflection during the research process. Reflexive thematic analysis [69] and the IBPA Framework will be used by the research team to inform the analysis the qualitative data collected in Objective 4 to help capture and respond to the multi-level interacting social locations, forces, factors, and power structures that shape and influence the health of participants [55].

## Implications

This study builds on preliminary work by New Brunswick physicians *Losier *et al*.* that has shown that 40% of the ED visits of residents of special care homes in New Brunswick were determined to be potentially avoidable [66]. A more nuanced understanding of the experiences of older adults will help researchers critically assess often cited claims that inappropriate or preventable ED usage by older adults is due to issues such as poor care management, inadequate access to care, or poor choices on the part of patients [75]. Understanding the linkages between avoidable ED visits and the unmet care needs older adults from marginalized populations can help identify structural and systemic barriers in access to appropriate, continuous, and equitable care services. This study will offer unique insight into patients’, families’, and care providers’ lived experiences and perspectives on whether more integrated and accessible community services could help reduce ED visits in this community-dwelling older adult population in addition to offering suggestions of what a more equitable way of offering care might look like. These findings could help explain why older adults, including those from marginalized groups, frequently transition through the ED as they move along the health care continuum with the hope of smoothing that transition in the future by informing policy and clinical practice decision making at the institutional, regional, and provincial levels. Better use of EDs may also result in more efficient use of the health care system by allowing ED resources to be more closely targeted to those who appropriately require them.

## Discussion

This study’s design using EDs as sites for recruitment is based on the success of previous research using this method with a similar population of older adults with complex care needs in comparable ED settings [76]. The context of the post-pandemic state in which we are working has raised many barriers to developing a feasible plan for recruitment and data collection within the ED related to the possible return to restrictions to researchers’ access to EDs during future waves of COVID-19 and the extreme lack of human resources in the ED severely limiting the abilities of members of the care team to assist with participant recruitment. After extensive consultation with managers at each ED site, the research team began with a passive recruitment strategy to minimize the impact on the care team working in the EDs and with a plan to conduct all data collection off site. However, this strategy did not work well with only two research participants recruited over 4 months, so the research team engaged a research assistant to assist with active recruitment at the two ED sites. To mitigate possible issues related to a lack of engagement of clinical staff in the project [77], we have engaged both emergency physicians and nurse managers at both ED sites in the development of the recruitment plan enabling its customization to work as smoothly as possible at each site. Drawing on recommendations from the literature [78], the research team has implemented measures to address the creation of a research-friendly culture in the ED such as: including the ED’s Research Director on the study’s research team and establishing good rapport between researchers and clinical staff using nurse managers as a key contact point. The recruitment methods and data collection tools have been carefully designed and customized to each site to avoid any unnecessary burden on members of the care team working in the ED during the recruitment phase of the project. The passive recruitment strategy tried initially resulted in insufficient recruitment necessitating the adoption of a more active recruitment plan involving the use of an RA in the EDs to help recruit participants. To do so, additional barriers related to the PI’s positionality as a postdoctoral fellow and institutional challenges related to the funding and logistics required to hire an RA to assist with this project had to be overcome. The current state of ED overcrowding has created challenges in other research projects resulting in a lack of face-to-face communication between researchers and eligible patients [79]. When preparing a plan for data collection, the research team anticipated difficulties accessing private spaces for a sufficient amount of time needed for both informed consent and data collection. The lack of private space combined with concerns over self-administering the surveys due to low literacy rates of patients contributed to the team’s decision to recruit only in the ED with data collection being done later virtually after the patient leaves the hospital.

### Limitations

We know that many older adults who encounter barriers to care frequently use many different health and social services, and that ED visits are a good proxy of this use. However, not all older adults who experience challenges accessing care are frequent ED users so we might miss some patients by recruiting in an ED. We might experience lower uptake of patients in the ED who are willing to participate in the study because of recruitment taking place in the ED where patients might be feeling unwell and perhaps not interested being recruited to research project at this time, or did not see, or could not read, our recruitment flyer. If eligible participants do not contact the PI while they are waiting in the ED, they might misplace the tab with the PI’s contact information or forget to contact the PI once they leave the ED. Furthermore, we might miss a subset of patients who cannot contact us upon leaving the ED due to being precariously housed or not having reliable access to the internet or a phone. Also, the sample used in this study will not be random and thus might be affected by selection bias including participants with more polarized perspectives than the general population. As such, generalizations from this study cannot be drawn to all community dwelling older adults with high ED use from historically marginalized groups. To address this concern, we can use maximum variation sampling to enhance diversity if a large enough group of potential research participants exists. We will also use member-checking to ensure that our findings resonate with the lived experiences of patients and family care partners to help ensure reliability and transferability to other contexts. We are also replicating this study at the Montfort Hospital in Ottawa, Ontario to help determine if the findings are generalizable outside of Atlantic Canada.

## Data Availability

Not applicable.

## Abbreviations

ED: Emergency Department
IBPA: Intersectionality-Based Policy Analysis Framework
iKT: Integrated Knowledge Translation
MSC: Most Significant Change
PHS: New Brunswick Health Council’s Primary Health Survey 2020
RA: Research Assistant
